# 

**Published:** 1889-09-28

**Authors:** 


### NOTICE TO CORRESPONDENTS.

All MS., Letters, Books for Review, and other matters intended for the Editor, should be addressed The Editor, The Lodge, Porchester Square, London, W.

### Notice to Advertisers and Subscribers.

All Advertisements, Orders for Copies of The Hospital, and Business Communications should be addressed to The Publisher, *not to the Editor*, The Hospital, 139-140, Salisbury-court, Fleet-street, E.C.

Vol. V., now ready, 4s., post free. Cases for binding, may be had of the Publisher, at 1s. 6d. each.

### To Residents, Secretaries, and Matrons.

The Editor will be glad to have the Regulations and each Report as issued by Asylums, Hospitals, Convalescent and Nursing Institutions, as well as those of other Charities, and an early copy in duplicate of all Appeals and Festival Notices, etc., addressed to *The Editor, The Lodge, Porchester-square, London, W.* Any superintendent, secretary, matron, or other chief official who regularly sends such information may be registered, on application to the Publisher, to receive free of cost a cover or binding The Hospital in half-yearly volumes.

### Amusements and Relaxation.

### SPECIAL NOTICE.

**ACROSTIC COMPETITION.**

**The Prize Editor having been requested by several correspondents to continue the ORIGINAL and SET Acrostic Competitions will be glad to receive the names of all intending competitors on or before October 3rd.**

### Second Quarterly Word Competition Commenced July 6th, ends September 28th.

Three Prizes of 15s., 10s., 5s., will be given for the largest number of words derived from the words set for dissection.

Proper names, abbreviations, foreign words, words of less than four letters, and repetitions are barred; plurals, and past and present participles of verbs, are allowed. Nuttall's *Standard* dictionary only to be used.

N.B.—Word dissections must be sent in WEEKLY, arranged alphabetically, with correct total affixed.

The word for dissection for this, the *Thirteenth* week of the quarter, being "DOVER."

**Results of Eleventh Week.**

| Names. | Sept. 20th. | Totals. | Names. | Sept. 20th. | Totals. |
|---|---|---|---|---|---|
| Nurse Duty | 426 | 981 | F. C. J. | - | 305 |
| Jumps | — | 30 | Percy Verance | — | 18 |
| N'importe Qui | 273 | 778 | Gleniffer | — | 175 |
| Tinie | — | 186 | Gypsye | 388 | 878 |
| Amicus | — | 430 | Ladyr | — | 127 |
| Patience | 436 | 991 | A. B. | — | 170 |
| Broxbourne | — | 398 | Essie | — | 393 |
| Speedwell | 360 | 864 | Cowboy Bill | — | 179 |
| Lightowlers | — | 356 | M. L. A. | — | 117 |
| Lauriston | — | 345 | W. G. R. | 331 | 759 |
| Sister | 372 | 893 | Little Tuppy | 149 | 259 |
| Rattler | — | 28 | Embryo | — | 56 |
| Paignton | — | 334 | Jenny Wren | 289 | 726 |
| Fassifern | — | 141 | Pearl | — | 132 |
| W. R. E. | 419 | 960 | Condorcet | — | 450 |
| Buxton | — | 167 | R. W. | — | 43 |
| Nux Vom | — | 263 | Electrician | 414 | 732 |
| Esperance | 415 | 966 | Nurse Amie | — | 89 |
| Spes | — | 24 | Sunshine | 407 | 630 |
| Secnarf | — | 229 | Juvenis | — | 23 |
| M. W. | 411 | 966 | Qu'appelle | 280 | 771 |
| Rover | — | 226 | K. Jarman | — | 43 |
| Leirion | — | 195 | Reveal | — | 196 |
| Matron | — | 174 | Amos | 224 | 224 |
| Isabel | — | — | | | |

**Notice to Correspondents.**

N.B.—All letters referring to this page which do not arrive at 139 and 140, Salisbury-court, London, E.C., by the *first post on Thursdays*, and are not addressed PRIZE EDITOR, will in future be disqualified and disregarded.

**Conditions.**

I. Every paper sent in must be clearly written, and one side only must be used. All competitors must mark at the head of their papers the number of their competition.

II. Each paper must be signed by the author with his or her real name and address. A *nom de plume* may be added if the writer does not desire to be referred to by us by his real name. In the case of all prize-winners, however, the real name and address will be published.

III. All communications relating to prize competitions should be addressed to "The Prize Editor, THE HOSPITAL, 139 and 140, Salisbury-court, London, E.C." Competitors are requested not to include in letters, etc., to the Prize Editor communications on matters of other business. All letters must be fully prepaid.

IV. The Prize Editor of THE HOSPITAL is the sole judge of the competitions, and his decision is in all cases final and without appeal.

V. The Prize Editor reserves the right to publish any paper sent in, whether it receives a prize or not. He does not hold himself responsible for any MSS. sent, nor can he undertake to return rejected competitions.

### Scraps and Gleanings.

OVER £30 was collected in Sidcup on Hospital Sunday.

SOME cases of cholera have occurred at Constantinople.

TUNBRIDGE WELLS subscribed £32 on Hospital Saturday.

THE death is announced of Dr. David Wilson, of Brook-street.

AT Tonbridge on Hospital Sunday the collection amounted to £42.

BROAD BLUNSDON collection on Hospital Sunday amounted to £1 odd.

THE London Temperance Hospital has received £200 from "H. W. V

A MAN died lately at Appledore from the effects of swallowing a needle.

MR. MANSELL-MOULLIN has been appointed surgeon to the London Hospital.

A SEASIDE Camp for 130 boys has been held successfully at Filey in Yorkshire.

A STATUE has been erected at Paris to Henri Bonley, the celebrated veterinary surgeon.

AN epidemic of scarlet fever has broken out amongst the school children of Greenock.

GRANTHAM HOSPITAL has opened its doors to members of friendly societies at half-price.

MR. GLADSTONE has given an iron building to Hawarden as a free library and reading-room.

The Queen has presented a coloured picture of herself on satin to the Station Hospital at Quetta.

THE British Home for Incurables has received £1,000 by the will of the late Mrs. Martin Bulmer.

DR. EAST APTHORPE has been dismissed from her Majesty's service with disgrace, for stealing £10.

SIR MORRELL MACKENZIE and Dr. Robson Roose are on the committee of the new Arts and Letters Club.

THE Empress Augusta has given £40 to the fund for the relief of the sufferers by the Antwerp explosion.

ST. GEORGE'S HOSPITAL received the flowers offered at Wootton Bassett service on Hospital Sunday.

DEVIZES COTTAGE HOSPITAL has benefitted to the extent of £22 by the late Friendly Societies' Demonstration.

THE rules and regulations of the Rawson Convalescent Home have been printed and passed by the committee.

WATFORD DISTRICT COTTAGE HOSPITAL reports that during the last year it has constantly had all the beds occupied.

BIRMINGHAM NEW INFIRMARY has been visited by Coventry Guardians, who contemplate a new infirmary to their workhouse.

THE death is announced of Dr. Alexander Thomson, of Edinburgh, famed alike for his medical and theological knowledge.

VIENNA hospital for infectious diseases has been a fortnight without inmates; such a thing has not happened for many years.

FORTY-SIX patients of Salisbury Infirmary have, during the last year, been permitted to make use of the Turkish baths in the town.

STRATFORD-ON-AVON has treated 202 in-patients and 509 out-patients last year. The annual meeting was held in the end of August.

KIRKINTILLOCH boasts a very successful class on "First Aid," the lecturer being Dr. Martin, of the St. Andrew's Ambulance Association.

CORK has only been able to screw £300 out of the Town Council for its infectious hospital, and it is feared the institution must close its doors.

APPLICATIONS for the post of surgeon to Victoria Infirmary, Glasgow, having been so numerous, the question has been deferred to the next quarterly meeting.

ST. MARK'S OPHTHALMIC AND AURAL HOSPITAL, Dublin, makes a special appeal for funds. Over a hundred out-patients are sometimes treated in one day.

THE death is announced of Dr. G. W. Royston Pigott, M.D. and F.R.S. He practised at Harrogate, and distinguished himself by his researches in microscopy and astronomy.

AT a meeting of the managers of the Aberdeen Dispensary it was intimated that Dr. Francis Edmond, of Kingswells, had given a donation of £300 to the funds of the institution.

CHOLERA has made its appearance in the neighbourhood of Kerind, near the Western frontier, having been imported from Bagdad. Five deaths, says a Reuter's telegram, are occurring daily.

LAST week the mortality in Glasgow was at the rate of 20 per 1,000 of the population per annum, as compared with 21 in the previous week, and 17, 19, and 24 in the corresponding periods of the three preceding years.

DAUCHEZ noticed that in mumps contagion took place in three cases, after an interval of fifteen days—once seventeen days—after apparent infection. Dr. Dauchez concludes that the incubation period is fifteen days on an average.

IT was reported to the Public Health Committee of Edinburgh Town Council last week that there were just now 167 patients in the City Infectious Hospital, of whom 84 are under treatment for scarlatina. Campsie House has 18 convalescent patients.

THE late Miss Winlaw has bequeathed the following donations: Aberdeen Royal Infirmary, £2,000; Aberdeen Incurable Hospital, £2,000; Aberdeen Blind Asylum, £2,000; Aberdeen Deaf and Dumb Institution, £1,000; Aberdeen Sick Children's Hospital, £500; Jewish and Foreign Missions, £2,000.

AT Bridge of Allan the other day a cat gave birth to four kittens, three alive; the fourth was dead, unfortunately, for had it lived it would have been (as a show) a fortune to even a Scottish Barnum. It has seven legs and two tails, more curious than the cat of old, which was said to have had nine tails.

The Rev. Gethin Davies, D.D., Llangollen, Principal of the North Wales Baptist College, has received a cheque for £20,000 from Alderman Cory, J.P., of Cardiff, as an endowment for the theological chair at the Llangollen College. Alderman Cory has also promised a further donation of £2,000 to the home mission funds connected with the college.

